# Cyanine-5-Driven Behaviours of Hyperbranched Polymers Designed for Therapeutic Delivery Are Cell-Type Specific and Correlated with Polar Lipid Distribution in Membranes

**DOI:** 10.3390/nano11071745

**Published:** 2021-07-02

**Authors:** Joshua D. Simpson, Denni L. Currin-Ross, Gayathri R. Ediriweera, Horst Joachim Schirra, Nicholas L. Fletcher, Craig A. Bell, Maria C. Arno, Kristofer J. Thurecht

**Affiliations:** 1 Australian Institute for Bioengineering & Nanotechnology, University of Queensland, St. Lucia, QLD 4072, Australia; j.simpson@uq.edu.au (J.D.S.); a.ediriweera@uq.edu.au (G.R.E.); n.fletcher1@uq.edu.au (N.L.F.); c.bell1@uq.edu.au (C.A.B.); 2 ARC Centre of Excellence in Convergent Bio-Nano Science & Technology, ARC Training Centre for Innovation in Biomedical Imaging Technology, University of Queensland, St. Lucia, QLD 4072, Australia; 3 Centre for Advanced Imaging, University of Queensland, St. Lucia, QLD 4072, Australia; d.denni@uq.edu.au (D.L.C.-R.); h.schirra@uq.edu.au (H.J.S.); 4 School of Environment and Science, Griffith University, Nathan, QLD 4111, Australia; 5 Griffith Institute for Drug Discovery, Griffith University, Nathan, QLD 4111, Australia; 6 School of Chemistry, University of Birmingham, Edgbaston, Birmingham B15 2TT, UK; m.c.arno@bham.ac.uk; 7 Institute of Cancer and Genomic Sciences, University of Birmingham, Edgbaston, Birmingham B15 2TT, UK

**Keywords:** nanomedicine, polymers, endosomal escape, fluorescence, cellular uptake, trafficking, materials design

## Abstract

The ability to predict the behaviour of polymeric nanomedicines can often be obfuscated by subtle modifications to the corona structure, such as incorporation of fluorophores or other entities. However, these interactions provide an intriguing insight into how selection of molecular components in multifunctional nanomedicines contributes to the overall biological fate of such materials. Here, we detail the internalisation behaviours of polymeric nanomedicines across a suite of cell types and extrapolate data for distinguishing the underlying mechanics of cyanine-5-driven interactions as they pertain to uptake and endosomal escape. By correlating the variance of rate kinetics with endosomal escape efficiency and endogenous lipid polarity, we identify that observed cell-type dependencies correspond with an underlying susceptibility to dye-mediated effects and nanomedicine accumulation within polar vesicles. Further, our results infer that the ability to translocate endosomal membranes may be improved in certain cell types, suggesting a potential role for diagnostic moieties in trafficking of drug-loaded nanocarriers.

## 1. Introduction

The emergence of nanotechnology for medical applications, nanomedicine, has been hailed as a significant improvement in many areas of medicine, particularly the treatment of solid tumours [1]. Through targeted design, sub-micron-sized, engineered nanoparticles can improve the biodistribution, stability, stealth, and tumour accumulation of loaded drug molecules [2]. The potential advantages conferred by nanomedical formulations have led to cancer treatment applications becoming a significant focus of the field [3]. Through improved site-specific deposition and subsequent chemotherapeutic delivery to cancerous tissue, it is possible to reduce toxic side effects and enhance therapeutic efficacy, especially in the case of fragile or insoluble molecules that are otherwise challenging to deliver. This approach has the potential to radically alter therapeutic regimes and improve patient quality of life [4].

At present, results in preclinical studies highlight the exciting potential of nanomedicines. However, despite the promise indicated, clinical translation remains a rare event [5,6,7]. With nanomedicine rapidly approaching the precipice of regular clinical translation, the success rate will likely hinge on the fundamental understanding of behaviours exhibited by each architecture and formulation on a case-by-case basis [8,9]. Materials and delivery systems possessing greater levels of characterisation and understanding of underpinning biological behaviours and interactions are theoretically more apt candidates for undergoing the transition into the clinical environment [9]. Thus, a great deal of research has begun to focus specifically on potential nanomedicine interactions with biological barriers [2,10]. The complex array of interactions between nanomaterials and biological phenomena yields numerous mechanisms that alter the in situ behaviours of materials [2,10].

Among these barriers, cellular-level interactions and the behaviours of materials within the cellular milieu have risen to prominence due mainly to the development of environmentally-sensitive materials that possess efficacy intrinsically tied to their intracellular trafficking [9,11]. While not well explored in terms of nanomaterials, these behaviours are likely cell-type dependent, as has been seen with other systems [12], with variance in cellular properties being tied to the tissue of origin [13,14], cellular function [15,16], and disease impacts [15,17].

Fluorophore selection [18,19], positioning [19], and concentration [20] have previously been determined to play a role in the outcome of interactions between polymeric nanocarriers and selected cell lines. Comparatively, in the case of established venom peptides, changes in cellular interactions have been tied to a combination of increased membrane interactions, the degree of disorder in the plasma membrane, and altered membrane permeability [12], which likely also apply to fluorophore-driven internalisation [21]. This suggests that enhanced membrane interactions likely increase translocation of small materials and increase their access to internalisation pathways [12,19].

Downstream behaviours such as endosomal escape [22] have also been demonstrated to have a membrane interaction component in both polymeric materials [23,24] and other systems [12]. This is of interest, as the induction of endosomal escape is widely considered favourable in improving therapeutic delivery and thus efficacy of polymeric materials for nanomedicine. Despite the widely acknowledged significance of this phenomenon, the processes involved and the cellular mechanics associated with this behaviour remain controversial, with several competing theories existing within the field [22]. Of particular note, membrane disruption during endolysosomal transit has been tied to membrane characteristics [12] and cell type [25]. It is essential to understand how design choices, such as fluorophore selection, may impact downstream functionality, such as endosomal escape.

Here, we explore fluorophore-driven internalisation across a selection of cell-lines using cyanine-5 (Cy5)-labelled hyperbranched polymers (HBPs) synthesised to yield controlled positioning of the probe either on the periphery through post-synthesis modification or within the hydrophilic core through co-polymerisation of fluorescent monomers [19]. We utilised these materials to assess cell-type susceptibility to Cy5-driven ingress and investigated differences in internalisation behaviours through a semi-quantitative region of interest (ROI) analysis. in order to draw more meaningful comparisons, we used principal component analysis (PCA) to probe the information gathered, identifying pertinent underpinning roles of materials design choices upon the behaviour of a model polymer assembly across several cell types. Our analyses identified three principal components representing ~97% of variance between samples and showed that these components can effectively discriminate between cell types. Through examining the ability of these polymers to induce endosomal escape using calcein assays in each cell line, we were able to assess the role of fluorophore placement on membrane-disruption in a cell-line dependent manner. Given that the behaviours described have been demonstrated to be tied to membrane composition and characteristics, we correlate the results of these findings to polar lipid distribution using the solvatochromatic lipid stain Nile red to label cholesterol-rich domains. In this fashion, we endeavoured to identify whether fluorophore-driven internalisation and endosomal escape behaviours of these HBPs are tied to inherent cellular membrane characteristics.

## 2. Materials and Methods

### 2.1. Polymer Synthesis and Characterisation

The synthesis and characterisation of polymeric materials used in this study have been previously reported [19]. The HBPs selected for this study contained Cy5 as either an internal monomer or as a chain-end modification. A fluorophore-free HBP derivative was produced through reversible addition-fragmentation chain-transfer (RAFT) of polyethyleneglycol monomethyl ether methacrylate (PEGMA) and ethylene glycol dimethacrylate (EGDMA), and the final materials were isolated and purified by preparative size exclusion chromatography (SEC, Cytiva ÄKTA, Uppsala, Sweden) and characterised using nuclear magnetic resonance (NMR, Bruker Biospin, Rheinstetten, Germany) spectroscopy and gel permeation chromatography (GPC, Waters, Milford, MA, USA).

### 2.2. Cell Culture

Several cell lines were maintained in either Roswell Park Memorial Institute (RPMI) or Dulbecco’s Modified Eagle Medium (DMEM) as per American Type Culture Collection (ATCC) instruction. This medium was obtained from Thermofisher and supplemented with penicillin, streptomycin, and glutamine, with foetal bovine serum being added at 10% volume/volume prior to use. For these studies, we examined two breast cancer cell lines (MDA-MB-231 and MDA-MB-468), an ovarian cell line (SK-OV-3), a glioblastoma cell line (U251-mg), and two healthy cell lines (NIH 3T3 fibroblasts and CHO-K1). These cell lines were stored at 37 °C with 5% CO_2_ in a water-jacketed incubator, being passaged as required. To minimise variance, all experiments were performed between passages 15–25.

### 2.3. Live Cell Confocal Microscopy

Cells were imaged using a Zeiss 710 laser scanning confocal microscope (Carl Zeiss Microscopy, Oberkochen, Germany) housed within the Australian National Fabrication Facility (ANFF) Queensland Node. This machine is equipped with a heated stage, 40 × water objective, a 405-nm diode, and helium-neon and argon lasers. Prior to imaging, 5 × 10^5^ cells in 1.5 mL of fresh medium were plated into slide-bottomed culture dishes (Mattek), returned to the incubator and left to grow overnight. The imaging conditions varied with the experimental applications as described below.

### 2.4. Internalisation Kinetics

In order to assess the influence of Cy5 positioning as a function of cell-type, the cells were washed with PBS three times, and the media was replaced with phenol red-free media. The cells were subsequently imaged to verify cell health. The selected HBP solution was then added to generate a final concentration of 100 µg/mL. During imaging, the Cy5 derivatives were excited at 633 nm, and the detection range was set to 641–700 nm. Images of cell populations were taken at 5, 10, 15, 30, 45, 60, and 90 min. To garner internalisation rate information, the region of interest tool in Zen Blue Zeiss Lite (Carl Zeiss Microscopy) was used to assess the fluorescence intensity within the intracellular space and the surrounding extracellular medium within images. The values were exported to Microsoft Excel, the ratio of the former values divided by the latter was determined, and the average and standard deviation of these values were ascertained for each time-point. This information was exported to Prism 8 (Graphpad) and plotted as line graphs.

### 2.5. Calcein Assay

For endosomal escape assays, calcein was dissolved in phenol red-free RPMI (Thermofisher, Waltham, MA, USA) or Opti-MEM (Thermofisher) to make 100 µg/mL solutions. At 4 h prior to imaging, cells were washed with PBS three times and 1 mL of the suitable calcein media added to the imaging dish before being returned to the incubator for 2 h. After this time elapsed, the cells were washed with PBS three times and the media replaced with phenol red-free media containing 100 µg/mL of the selected HBP (or media alone for control samples). Once exposed to this solution, the cells were again returned to the incubator for an additional 2 h. Populations of cells were then imaged using sequential scanning, calcein being excited with 488 nm excitation and its emission being collected between 500–580 nm. The settings for Cy5 HBPs were as described prior. For population images, the cells were counted and calcein fluorescence classified as either punctate or diffuse for each cell. These data were recorded in Microsoft Excel. Endosomal escape efficiency was then calculated by dividing the population defined as diffuse by the total population, whereby these results were then transferred to Prism 8 for graphing.

### 2.6. Nile Red Staining

To ascertain whether a relationship exists between vesicle polarity and endosomal escape behaviours, cells were stained with Nile red (Sigma-Aldrich, St. Louis, MO, USA) as per supplier protocol and imaged using 488 nm excitation to favour the blue-shifted fluorescent species, with two detectors used to identify the polar and non-polar staining of lipids. To better visualise differences in the distribution of lipid populations, images were exported as single channels and imported into ImageJ. Using the image calculator plugin, the blue-shifted channel was divided by the standard Nile red channel in order to produce a ratiometric image. Images were assessed for plasma membrane and vesicular membrane values. These images were exported as TIFF files, and the values retrieved from ROI assessment were recorded in Microsoft Excel. These data were then transferred to Prism 8 for graphing.

### 2.7. Principal Component Analysis of Rate Kinetic Data

In order to ascertain whether Cy5 HBPs and cell types differ by given time points, a principal component analysis (PCA) was performed. Because the kinetic rate data here are pooled cross-sectional data (i.e., observations that come from different cells across the time series), arbitrary samples from neighbouring time points can be paired to construct a time series row in the data matrix. Thus, a permutation analysis was initially undertaken to determine whether the arrangement of the samples in the data matrix would be a significant factor in the outcome of the PCA. The data matrix was scaled to unit variance but not mean centred (UVN scaled) for the subsequent analysis.

Two hundred permutations of the columns in the rate kinetic data matrix were generated. Principal component analysis was then performed in RStudio (RStudio, Inc., Boston, MA, USA) on each permutation using the R package *pcaMethods*. To evaluate model quality for each PCA, the R^2^X and Q^2^ values were extracted. The R^2^X is the fraction of the variance of the X variables explained by the model, and Q^2^ is the predictive ability parameter of the model, which is estimated by cross validation. Histograms of the R^2^X and Q^2^ values from the 200 permutations were plotted overlaid with its density curve (Figure S1). No significant impact of the permutations was noted, with both the R^2^X and Q^2^ values exhibiting a narrow peak.

The single permutation that had an R^2^X and Q^2^ value nearest to the maximum value from the histograms, and thus the most probable outcome, was chosen for the final analysis. The chosen permutation was exported into SIMCA 16 (Sartorius Stedim AB). An initial PCA was performed with three components selected, which attained ~97% of the variability. Data from the U251-mg cell type were subsequently removed from further analysis as these cells exhibited a large deviation from the rest of the samples/groups. Two additional PCAs were generated to characterise cell-type effects, one each for the internal and external HBPs, respectively. The scores and loadings plots were used to interpret the PCA models. See Supplementary Table S1 for the figures of merit of all PCA models.

### 2.8. Correlation Analysis

In order to determine whether relationships exist between Nile red lipid staining and improvements in endosomal escape, a correlation was assessed through graphical means. This was achieved using ROI data obtained from vesicles within the ratiometric images and plotting the average polar:non-polar ratio against improvement in endosomal escape and comparing to a linear fit in Prism 8.

## 3. Results

### 3.1. Cy5-Labelled HBP Syntheses and Characterisation

**Figure 1:**
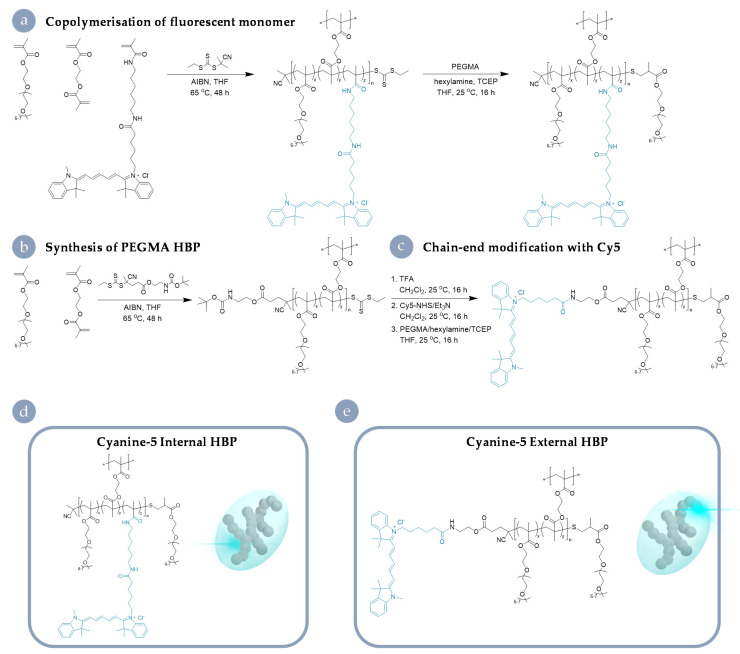
Synthesis of Cy5 HBP analogues. (**a**) Co-polymerisation of Cy5-labelled monomers to produce the Cy5 internal HBP. (**b**) Synthesis of a base PEGMA architecture and (**c**) post-modification with Cy5 NHS to yield the external derivative. Final macromolecular architectures with the fluorophore positioned within the hydrophilic corona (**d**) and towards the periphery (**e**).

The full details of materials synthesis and the results of characterisation are available elsewhere [19]; however, a summary table of important physicochemical properties is available in the supplementary information (Table 1). In short, RAFT polymerisation was used to produce branched polymers comprised primarily of PEG to represent an archetypical biocompatible nanomaterial of ~10 nm in size. These materials are based on a well-established class of hyperbranched polymeric materials previously utilised for preclinical imaging and therapeutic delivery [26,27,28,29]. For the HBPs used in this study, Cy5 labelling was achieved through simple covalent attachment chemistries. One analogue (Cy5 internal) was produced through statistical incorporation of a fluorescent monomer into the PEGMA hyperbranched structure. The other (Cy5 external) was manufactured through chain-end modification; a base HBP architecture was generated and then labelled through the attachment of a reactive dye species to a terminal amine on the α chain end of the PEGMA HBP. Important for the generality of our findings, both approaches are well established and in widespread use for the attachment of imaging modalities and therapeutic molecules in branched and dendritic particles (Figure 1). In both cases, the dye was attached to the polymeric architecture via an amide group. The resulting materials did not differ in size, surface charge, or dye-loading. As such, the behaviours described herein are on account of the degree of shielding provided by the dye’s positioning on the macromolecular structure. These HBPs serve as an example system, being reflective of a number of materials in clinical trials [30]. To examine the role of the incorporation method of components and the consequent macromolecular structure plays in determining biological outcomes, we interrogated a suite of different cell lines following exposure to both analogues.

### 3.2. Uptake Behaviours of Cy5-Labelled HBPs in Different Cell Lines

**Figure 2:**
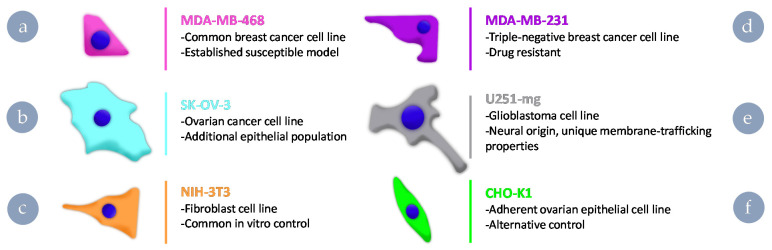
Cell lines selected for exploration of dye-mediated uptake and rationale for their inclusion in the study. (**a**) MDA-MB-468, (**b**) MDA-MB-231, (**c**) U251-mg, (**d**) SK-OV-3, (**e**) NIH-3T3, and (**f**) CHO-K1.

To assess the impact of dye-labelling strategy, we chose a number of cell lines commonly utilised in cancer nanomedicine in addition to two healthy cell controls (Figure 2). The two breast cancer cell lines were selected, as one is known to be susceptible to fluorophore impacts (MDA-MB-468) [19], whereas the other is a model commonly selected for drug resistance (MDA-MB-231). U251-mg was selected as, being of brain origin, it should possess distinct behaviours associated with endocytic pathways and trafficking. The ovarian cancer cell line SK-OV-3 was selected to pair with the drug-resistant breast cancer cell line and serve as a cancerous counterpart to non-cancerous ovarian control cells. A fibroblast cell line (NIH-3T3) frequently used as a control cell line for many applications was selected in conjunction with an ovarian epithelial cell line (CHO-K1) because the former may possess different endocytic behaviours on account of fibroblasts being able to fulfil a scavenger role. We aimed to give insight into the importance of considering the biological relevance of selected models for in vitro testing through screening across these cell lines. By treating cells within the population as a simplified one-compartment model, i.e., an external and internal compartment with defined rates of exchange between them, a semi-quantitative indication of ingress rate can be obtained ratiometrically.

**Figure 3:**
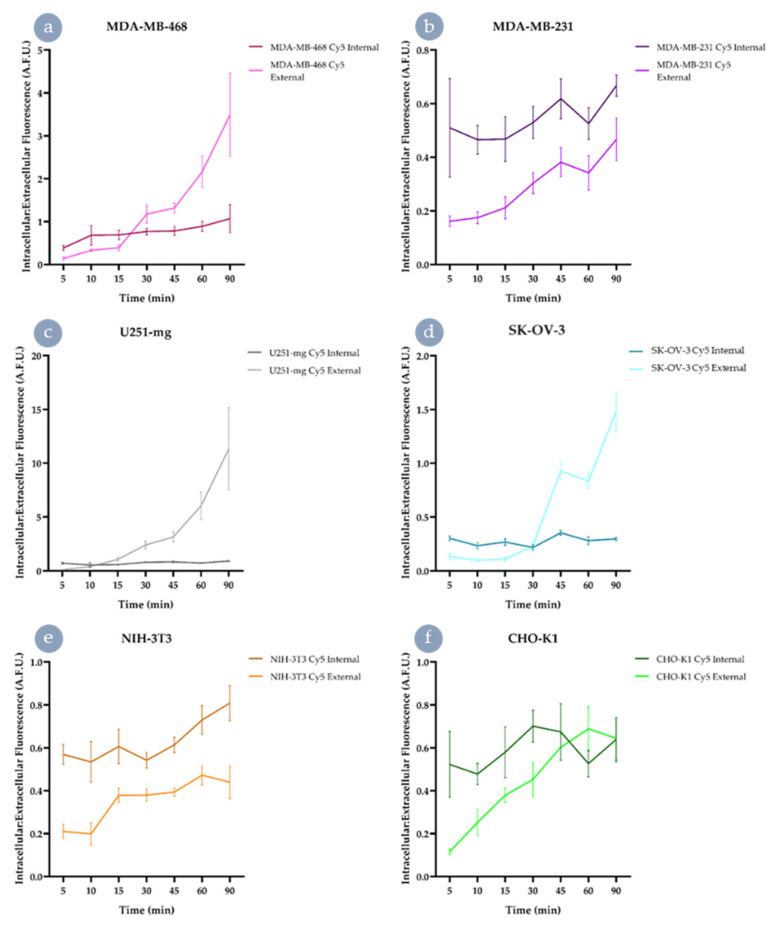
Uptake of Cy5 HBPs in different cell lines through dynamic live-cell imaging. The intracellular:extracellular ratio of Cy5 fluorescence for both Cy5 Internal and Cy5 External was assessed for (**a**) MDA-MB-468, (**b**) MDA-MB-231, (**c**) U251-mg, (**d**) SK-OV-3, (**e**) NIH-3T3, and (**f**) CHO-K1. Dark colours indicate Cy5 Internal and light Cy5 External; scale bars indicate ± a standard deviation.

Upon exposure to the Cy5 HBP analogues, differences in uptake behaviours appeared rapidly and were obvious (Figure 3). In MDA-MB-468, SK-OV-3, and U251-mg cell lines, Cy5 external HBP accumulated to a much greater extent within the cell interior than in the case of the drug-resistant MDA-MB-231 or control cells. Although SK-OV-3 exhibited a significant improvement in intracellular: extracellular fluorescence ratio for Cy5 external, this increase was significantly less than the other two cell lines observed to possess improved internalisation (U251-mg and MDA-MB-468). In comparison to the other cell lines, MDA-MB-231 showed significantly less internalisation in the case of the external derivative when compared to the internally labelled analogue. Interestingly, the fibroblast cells behaved similarly to the MDA-MB-231s; however, the epithelial cell line (CHO-K1) showed no significant difference in accumulation between the two Cy5-labelled HBP analogues from 45 min onwards. In comparison to the Cy5 external analogue (Figure S2a), the Cy5 internal exhibited far less difference between accumulation behaviours within the cell interior (Figure S2b), with some statistically significant differences at time points (e.g., 60 min), but very little by the end of the observation period. These results highlight the need to ensure suitable control cells are chosen for a desired biological application. For example, the NIH-3T3 fibroblast line is commonly used as an example of a control or healthy cell line for assessing association, internalisation, and toxicity of materials. However, this study indicates that these cells may be more resistant to materials of certain physicochemical properties than would be a cell more reflective of a cell type present in healthy tissue that may encounter the vector, e.g., epithelial tissue adjacent to the tumour mass.

To further interrogate the data obtained beyond obvious differences in ratio and begin to disentangle the factors that give rise to the impacts described, we explored these internalisation rates using principal component analysis (PCA).

### 3.3. Distinguishing Underlying Factors through Principal Component Analysis

**Figure 4:**
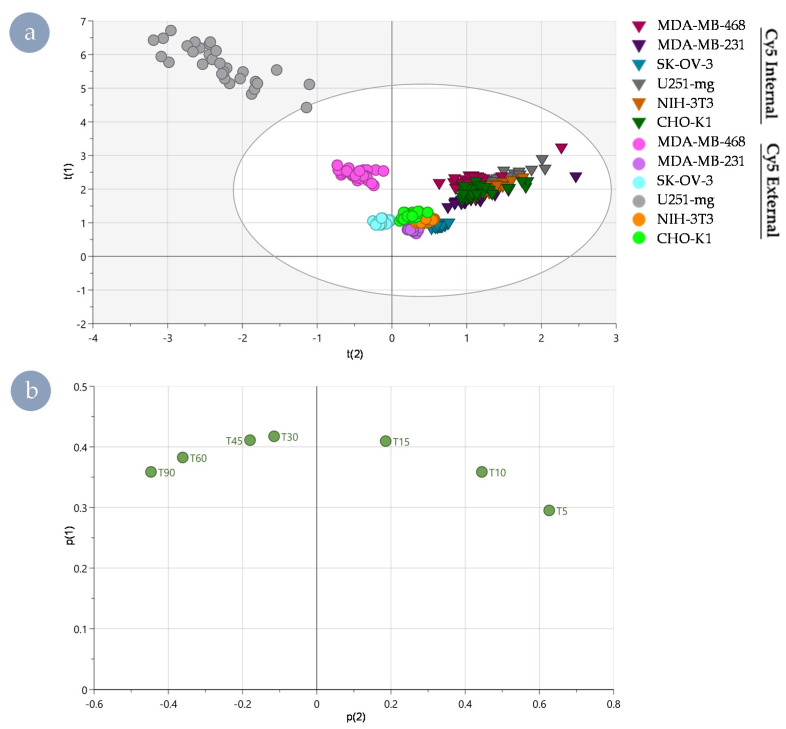
Separation of polymer analogues and cell lines by principal component analysis of internalisation rate data. (**a**) Scores plot of principal component 2 vs principal component 1 showing all cell lines, with polymer separation from component 2, whereas cell line is better identified using component 1. Internal Cy5 HBP: triangles; external Cy5 HBP: circle. (**b**) Associated loadings plot showing that polymer variance is best described by very early and very late time points and mid-time point data best separate cell lines.

A global principal component analysis (PCA) of the internalisation rates of all cell types showed clear intrinsic differences between the internal and external polymers and differences between the different cell lines. Apparent differences along t(2) were noted in the scores plot between the internal and external Cy5 HBPs (Figure 4a), driven by the initial (5 min) and late (90 min) time points in the corresponding loadings plot (Figure 4b). As such, these time points characterise the bulk of the resulting separation. This global PCA also highlighted that separation was more easily distinguished in the case of the external Cy5 HBP. In contrast, the separation between cell lines exposed to Cy5 Internal HBP is much less pronounced and yielded significantly less separated populations. Having been selected as a control cell line to ensure distinct species were present in the analysis, U251-mg dominated the variance observed in the results (Figure 4a).

**Figure 5:**
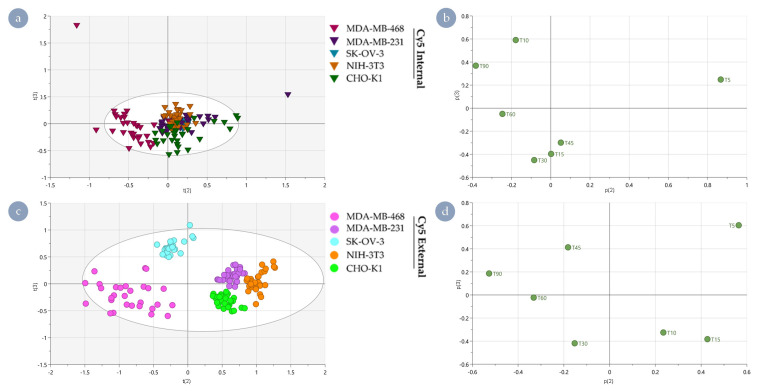
Separation of cell type and polymer species through principal component analysis with U251-mg excluded. (**a**) Scores plot of Cy5 Internal data only. (**b**) Corresponding loadings plot showing that 10- and 30-min time points are the main drivers of separation in the t(3) component, whereas 5 and 90 min predominantly influence the t(2) component. (**c**) Scores plot of Cy5 External data. (**d**) Corresponding loadings plot showing that 5, 15, and 90 min are the key drivers of separation along t(2), and 5 and 30 are important to separation along t(3).

While useful to validate that dramatic differences are easily detected utilising this technique, due to the likely significant differences in internalisation, trafficking, and secretory pathways as a result of its neural origin, U251-mg was excluded in further analyses to avoid obscuring nuanced differences between other cell lines (Figure 5). Further, both the kinetic rate data (Figure 3) and the results of the global PCA (Figure 4) exhibited apparent differences between Cy5 HBPs based on fluorophore positioning. To better explore cell-type dependency of fluorophore-driven behaviours, creation of sub-models for the internal and external Cy5 HBP analogues were established.

In the case of Cy5 Internal (Figure 5a), cell type separation is less pronounced compared to Cy5 External (Figure 5c). Of particular note, MDA-MB-468 can be distinguished in t(2) from all other cell types, driven via differences at time points 5 and 90 min. CHO-K1 can be distinguished from NIH-3T3 and MDA-MB-231 via differences in t(3), driven by time points at 10, 15, 30, 45, and 90 min. Interestingly, SK-OV-3 can be distinguished individually through t(1) (Figure S3) due to cumulative impacts from all time points.

Nanomedicine interactions with all cell types can be clearly distinguished from each other for Cy5 External (Figure 5c). Cell types MDA-MB-468 and SKOV3 can be distinguished from MDA-MB-231, NIH-3T3, and CHO-K1 in t(2), driven via predominant differences at time points 5 and 90 min. These two factors indicate that the underlying behaviours are driven by an initial susceptibility and the overall accumulation within the cell interior. These time points signify the ability of Cy5 to cross the plasma membrane and the ability of the cell line to mitigate entry in response. The time points 5, 10, 15, 30, and 45 min drive the separation of MDA-MB-468 and CHO-K1 from MDA-MB-231 and SKOV3 (both drug-resistant) along t(3). This suggests some of the mechanisms associated with drug resistance may be constitutively active and relevant to controlling Cy5-mediated ingress.

Despite obviously being due to very different mechanisms, similarities between the two sub-models in how the cell types are separated, especially with respect to MDA-MB-231 and NIH-3T3 clustering together, highlights that a proportion of the effects occurs regardless of fluorophore positioning within the macromolecular architecture. An important observation arising from this study is that the variance being assessed is very likely driven in part by inherent properties of the cell lines selected rather than specific physicochemical properties of the nanomaterial.

Given the results obtained, the cell type specificity identified likely represents a combination of basal metabolism and the susceptibility of cells to Cy5-driven membrane translocation. Cell-membrane characteristics are known to be highly cell-type specific. Variable aspects, such as rigidity [32] and membrane order [12], have previously been identified as important factors associated with translocation of macromolecules across the cell boundary, and permeation of this cellular feature is likely tied to lipid profiles and receptor density. In combination, the rate kinetics and global PCA results highlight that the inherent characteristics of biological model systems can yield significantly different outcomes in the assessment of nanomedicine internalisation behaviours.

Differences in internalisation profiles, timing of behaviours identified, and differing susceptibility of each cell line indicate an integral role of membrane characteristics. These observations raise the interesting possibility of these materials possessing different downstream behaviours pertinent to the delivery of therapeutics. This prompted us to explore whether the observed impacts of fluorophore positioning within the macromolecular architecture continue to yield cell-type dependent differences post-internalisation when interacting with trafficking membranes downstream.

### 3.4. Endosomal Escape Behaviours of Cy5 HBPs across Cell Lines

**Figure 6:**
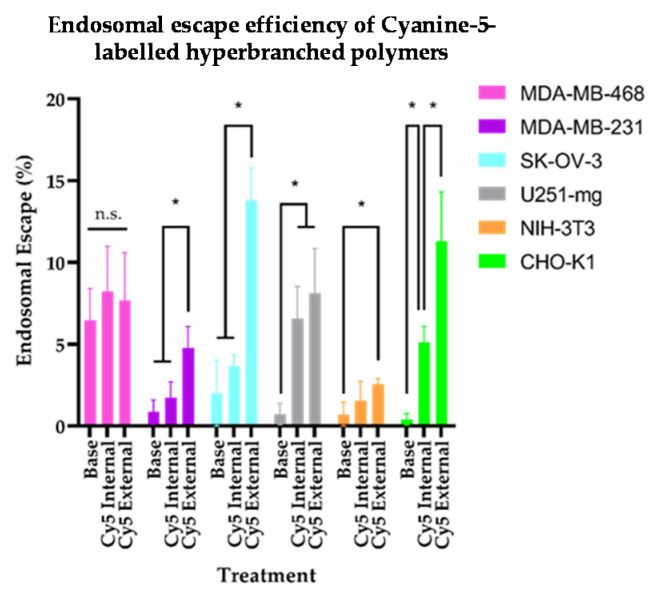
Endosomal escape properties of the assessed cell lines, with bar graph depicting endosomal escape percentage in untreated cells (base), cells exposed to the internal Cy5 analogue (Internal), and external Cy5 derivative (External). n.s.—no statistical difference, *p*-value > 0.05; *—*p*-value < 0.05.

The ability of materials (e.g., peptides [12]) to translocate endosomal membranes has been correlated with membrane characteristics that favour escape from trafficking vesicles and are thus tied to subsequently improved access to the cytoplasm. The ability to circumvent the vesicular membrane is of great importance to the delivery of therapeutics; as such, we examined the ability of the Cy5 HBPs to induce endosomal escape. The ability for each HBP analogue to interrupt vesicular membranes was assessed using a standard calcein assay [33]. Through this technique, a tracer dye passively labels endocytic compartments through internalisation along with extracellular medium. When small disruptions occur in the maturing trafficking vesicle, this allows the transfer of the impermeable, fluorescent small molecule to the cytoplasm, indicating that the compartment’s boundary has been compromised. The assays were initially performed with an exchange of media that did not contain any polymer derivatives to assess the general leakiness of endocytic compartments in each cell line to ensure that the observed results were not an intrinsic behaviour of the cell lines and were indeed due to interactions between the Cy5 HBP analogues and vesicular membranes. In terms of base endosomal escape (Figure 6), all cell lines exhibited some escape in the absence of HBPs; however, the percentage of the population was low enough to be statistically inconsequential except for the MDA-MB-468 cells. These cells exhibited a statistically significant background level of calcein release from the vesicular compartments and were also unique on exposure to polymers in that, regardless of the Cy5 HBP variant to which it was exposed, endosomal escape did not improve above the basal level. Each of the other cell lines assessed exhibited distinct endosomal escape behaviours in response to the positioning of Cy5 within the macromolecular architecture (Figure 6).

The two drug-resistant cell lines (MDA-MB-231 and SK-OV-3) exhibited no significant difference between their base rate of endosomal escape and that exhibited in response to when the dye was incorporated within the backbone of the architecture (Cy5 Internal). However, when the dye was attached to the HBP (Cy5 External) R group, a significant improvement in calcein release was observed. In the case of the U251-mg glioblastoma cells, the dye’s positioning within the polymer architecture did not matter, and both Cy5 Internal and Cy5 External imparted improved endosomal escape in comparison to the basal level. The NIH-3T3 cells appeared resistant to dye-induced endosomal escape, showing only a very modest increase in the case of Cy5 External. However, this was not the case for the internal analogue; notably, the fibroblasts exhibited the lowest percentage of endosomal escape in response to chain-end attached fluorophore of all cells. The healthy epithelial control cell line CHO-K1 demonstrated a distinct response to both Cy5 HBP derivatives, with dye-driven endosomal escape being most prominent when the fluorophore was attached to the periphery of the HBP architecture (Cy5 External). The differences between the NIH 3T3 fibroblasts and CHO-K1 epithelial cells again highlights the importance of selecting appropriate control cell lines for in vitro assessment, with the prior possessing the lowest percentage of escaped cells in response to Cy5 External of all the cell lines examined. This indicates that under certain circumstances, the use of NIH-3T3 fibroblasts in drug response and toxicity assays may underestimate the impact on healthy cells due to decreased endosomal escape and subsequent drug delivery. Further, the lack of correlation between ingress and endosomal escape as highlighted by the MDA-MB-468 cells showed no statistical improvement in response to any analogue, whereas cells resistant to fluorophore-mediated internalisation exhibited improved calcein release in response to the external derivative. As different cell lines possess a greater improvement in calcein release in response to HBP exposure (Figure 6) than those that exhibited increased internalisation rates (Figure 3), it is obvious that endosomal escape does not correlate with overall internalisation. These results suggest that downstream behaviours are not necessitated by overall accumulation behaviours but rather are driven by a separate underpinning biological susceptibility.

### 3.5. Both Internalisation Rates and Endosomal Escape Ability Are Correlated with Polar Lipid Distribution

**Figure 7:**
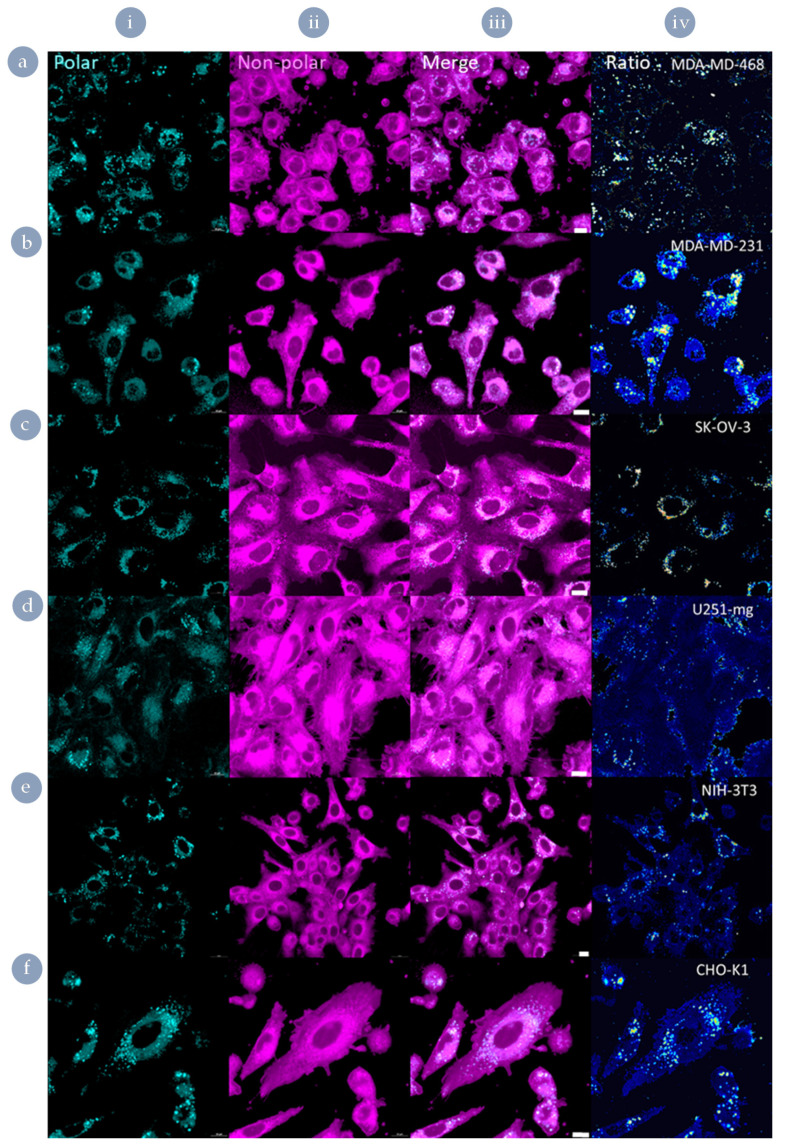
Characterisation of lipid distribution using Nile red staining. (**a**) MDA-MB-468, (**b**) MDA-MB-231, (**c**) SK-OV-3, (**d**) U251-mg, (**e**) NIH-3T3, and (**f**) CHO-K1; (**i**) polar lipid fluorescence channel, (**ii**) non-polar lipid fluorescence channel, (**iii**) merged, (**iv**) ratiometric images. Scale bars 10 µm.

To assess whether the different biological susceptibilities to internalisation and endosomal escape are tied to membrane characteristics, as has been reported for other systems [12], we assessed the cell lines’ lipid membrane polarity using the solvatochromatic dye Nile red (Figure 7). Due to the environmentally responsive spectral properties of this dye, when bound to polar lipids, the fluorescence undergoes a blue shift, meaning that areas rich in polar lipids (Figure 7 column i) can be distinguished from other lipid regions (Figure 7 column ii). This is achieved simply through imaging the different fluorescent species within Nile red stained populations and comparing the localisation between the two channels (Figure 7 column iii). However, to better facilitate comparison, the two channels can be expressed ratiometrically (Figure 7 column iv) to highlight areas that would otherwise be overlooked when assessed based on intensity images alone. At high magnification and zoom, discrete pockets of polar lipids can be seen in the ratiometric images of susceptible cell plasma membranes (Figure S4).

**Figure 8:**
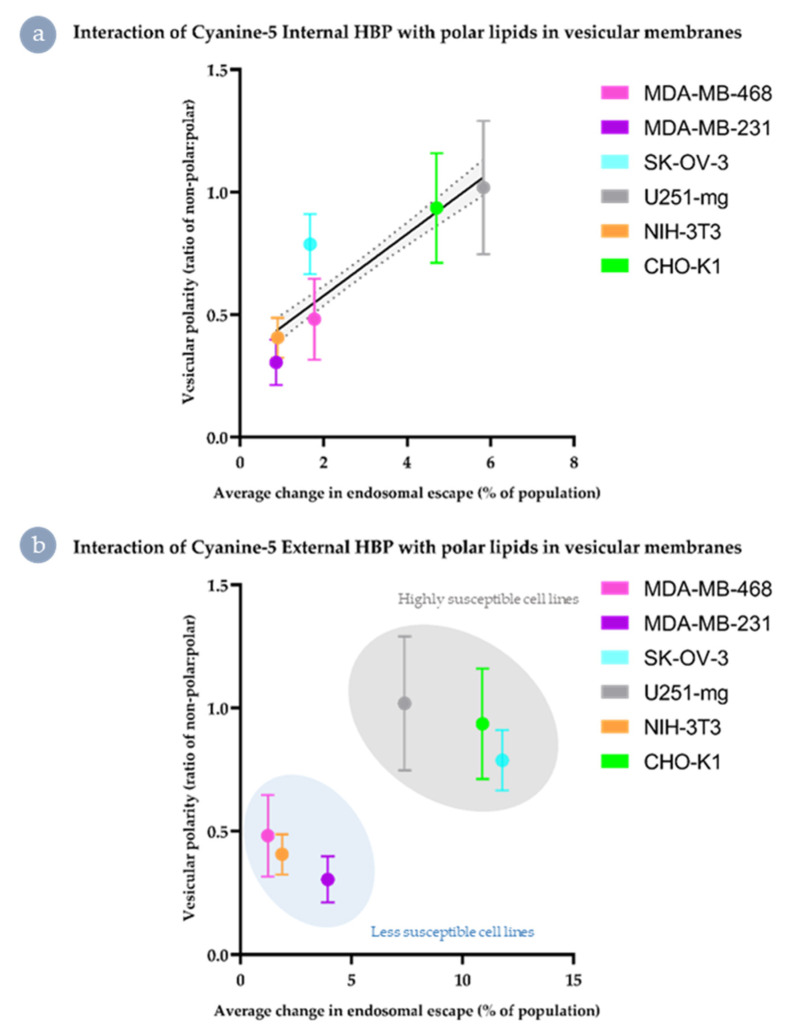
Correlation of polar lipid distribution with improved endosomal escape. (**a**) Cy5 Internal HBP (linear fit and 95% confidence interval indicated), (**b**) Cy5 External HBP (clustering indicated).

Polar vesicles were apparent within the ratiometric images of cell-lines susceptible to Cy5-driven internalisation of the external derivative (MDA-MB-468, SKOV-3, and U251-mg; Figure S4). However, qualitative differences were observed between these cell lines simply through the presence or absence of obviously highly polar regions (Figure S4). As such, a region of interest approach was performed to ascertain if there were nuanced differences between the vesicular membranes of the cell lines assessed that correlated with the endosomal escape behaviours observed (Figure 8). On average, no cell line produced a polar:non-polar ratio significantly higher than 10%, indicating that these differences are nuanced. However, these small variations in lipid composition correlate well with differences in endosomal escape behaviours.

In the case of Cy5 Internal (Figure 8a), there was a moderately linear relationship between vesicular polarity and improvement in endosomal escape, with the exception of the two drug-resistant cell lines (MDA-MB-468 and SK-OV-3). Comparatively, for the Cy5 External analogues, the small shifts in polar:non-polar ratio correlates to the separation of endosomal escape behaviours into two distinct populations (Figure 8b). This suggests that for the Cy5 External material, improvement in endosomal escape may effectively be tied to a threshold level of the proportion of polar lipids and cholesterol-rich microdomains in the maturing vesicular membrane.

## 4. Discussion

**Figure 9:**
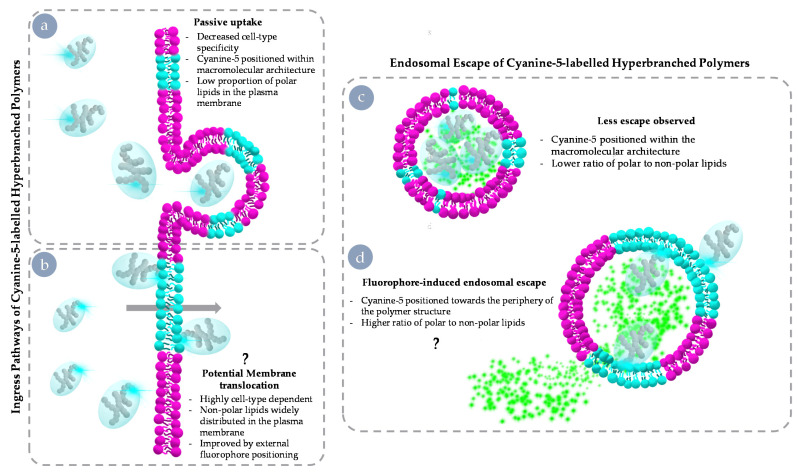
Schematic summary results and possible mechanism. Cy5-driven internalisation behaviours of HBPs (**left**). Passive uptake of Cy5 Internal HBP through being brought in with extracellular fluid (**a**) and potential membrane translocation of Cy5 External through a polar lipid-rich region of the plasma membrane (**b**). Fluorophore-driven endosomal escape behaviours (**right**); no escape of calcein exhibited when encapsulated in vesicles with low amounts of polar lipids (**c**); and membrane disruption resulting in calcein release enhanced by fluorophore positioning and polar lipid concentration (**d**).

The results presented demonstrate that fluorophore-driven impacts resulting from Cy5 incorporation into materials such as our HBPs are emphasised or ameliorated based on positioning within the macromolecular structure, yet those with strong interactions are also highly cell-type dependent. Differences in the weighting of these effects can be distinguished early on during observation and correlate well with membrane characteristics and the distribution profile of polar lipids (summarised in Figure 9). To summarise in brief, when fluorophore was positioned within the backbone of the polymer architecture and/or lipid membranes possessed low amounts of polar lipids, material was brought in with extracellular fluid (Figure 9a). When there were both pockets of polar lipids, and fluorophore was external to the macromolecular structure, direct permeation of the plasma membrane was more likely (Figure 9b). Downstream impacts on endosomal escape were mild (Figure 9c) unless the fluorophore had been attached to the periphery and maturing vesicles possessed a high degree of polar to non-polar lipids in their membranes (Figure 9d). These interactions likely underpin some of the unforeseen interactions exhibited in vivo and possess impacts across the spectrum of bio-nano interactions. While it has been previously reported that dyes such as Cy5 can interact with lipid membranes [21] and that this can be diminished through the use of sulpho-derivatives [18], the widespread use of Cy5 continues due to low cost, availability, optical properties, and utility. As such, it is vital to consider the potential impacts that the incorporation method may play in modifying interactions at the bio-nano interface, as those observed may not be reflective of the base platform being developed. It is imperative to consider the dye’s ability to impart potential interactions, such as π-π stacking and other aromatic interactions, that enhance transit across membranes through improved interaction with polar lipids and cholesterol.

Further, it would be salient to consider the potential impacts early in the design of a material. Although here we explored the impact of a far-red dye, the impacts described likely apply to a wide array of common additives or modular components of drug-delivery vectors, which is of import to polymeric materials in particular, as their modular nature and ease at which they can be modified could easily lead to oversight of such a subtle detail.

Poor practices, such as switching dyes or imaging modalities between applications, holds the potential to amplify misinterpretation of biological behaviours exhibited by nanomaterials in development for drug delivery, especially in the case of those designed for intravenous or systemic administration. Likewise, the results presented highlight the need to carefully consider the biological models utilised to assess interactions in vitro. For instance, when assessing treatment efficacy and cytotoxicity in vitro, selecting a tumour cell line such as U251-mg and a control cell line, e.g., NIH-3T3, may yield results that are heavily weighted towards showing selectivity and sensitivity regarding the cancerous cell line. This might not be the case if more realistic examples were selected for assessment. Where possible, control cells should be selected to reflect either the tissue of origin and/or healthy tissue adjacent to the tumour site in order to reduce the impact of unforeseen cell-type dependent interactions.

## 5. Conclusions

The impact of component-driven behaviours is an often-overlooked factor in the development of polymeric nanomedicines for drug delivery. Here, we describe the position-dependent influence of Cy5 across a range of cell lines. We identify that both polymer and cell type can be separated through principal component analysis of internalisation data. Further, although endosomal escape did not correlate with the internalisation results obtained, through imaging and assessment of Nile red staining in the cell lines observed, improved membrane translocation of externally labelled HBPs appears to be tied to the distribution of polar lipids, especially with regards to escape from vesicular compartments. These findings highlight the importance of the careful design of nanomaterials for drug delivery and the requirement for scrutiny of the biological models selected to assess bio-nano interactions.

## Figures and Tables

**Table 1:** Physicochemical properties of Cyanine-5 HBP analogues.

	Size (nm)	Labelling (a.u. per Particle) ^b^	ζ Potential (mV)	*M*_n,NMR_ (kDa)	*M*_n,SEC_ (kDa)	*Đ*_M_
Cyanine-5 internal HBP	7.5	0.17	−0.1	13.6	58.7	1.48
Cyanine-5 external HBP ^a^	7.1	0.29	−0.8	10.3	30.3	1.30

^a^ Data from unlabelled BOC-Amine Protected PEGMA HBP; ^b^ Based on the molar concentration of dye compared to the polymer concentration as determined by UV-Vis spectroscopy [31].

## Data Availability

Not applicable.

## Supplementary Materials

The following are available online at https://www.mdpi.com/article/10.3390/nano11071745/s1, Figure S1: PCA model validation; Table S1: Figures of merit for PCA model analysis; Figure S2: ROI kinetic rate data plotted by HBP analogue; Figure S3: PCA results of U251-mg excluded data; Figure S4: Polar lipid-rich membrane microdomains identified through ratiometric images.
