# Supplementary material for: Cyanine-5-Driven Behaviours of Hyperbranched Polymers Designed for Therapeutic Delivery Are Cell-Type Specific and Correlated with Polar Lipid Distribution in Membranes
Source: Nanomaterials (Basel). 2021 Jul 2;11(7):1745. doi: 10.3390/nano11071745 (PMC8308131; doi:10.3390/nano11071745)
Supplement: Supplementary file 1 [file nanomaterials-11-01745-s001.zip › nanomaterials-1236517-supplementary.pdf]

Supplementary Materials

Cyanine-5 Driven Behaviours of Hyperbranched Polymers Designed for Therapeutic Delivery are Cell-Type Specific and Correlated with Polar Lipid Distribution in Membranes

Joshua D. Simpson [1,2,3], Denni L. Currin-Ross [3], Gayathri R. Ediriweera [1,2,3], Horst Joachim Schirra [3,4,5], Nicholas L. Fletcher [1,2,3], Craig A. Bell [1,2,3], Maria C. Arno [6,7], and Kristofer J. Thurecht [1,2,3,*]

[1] Australian Institute for Bioengineering & Nanotechnology, University of Queensland, St. Lucia, QLD 4072, Australia; j.simpson@uq.edu.au (J.D.S.); a.ediriweera@uq.edu.au (G.R.E.); n.fletcher1@uq.edu.au (N.L.F.); c.bell1@uq.edu.au (C.A.B.)
[2] ARC Centre of Excellence in Convergent Bio-Nano Science & Technology, ARC Training Centre for Innovation in Biomedical Imaging Technology, University of Queensland, St. Lucia, QLD 4072, Australia;
[3] Centre for Advanced Imaging, University of Queensland, St. Lucia, QLD 4072, Australia; d.denni@uq.edu.au (D.L.C.-R.); h.schirra@uq.edu.au (H.J.S.)
[4] School of Environment and Science, Griffith University, Nathan, Qld 4111, Australia.
[5] Griffith Institute for Drug Discovery, Griffith University, Nathan, Qld 4111, Australia
[6] School of Chemistry, University of Birmingham, Edgbaston, Birmingham B15 2TT, UK; m.c.arno@bham.ac.uk
[7] Institute of Cancer and Genomic Sciences, University of Birmingham, Edgbaston, Birmingham B15 2TT, UK
* Correspondence: k.thurecht@uq.edu.au

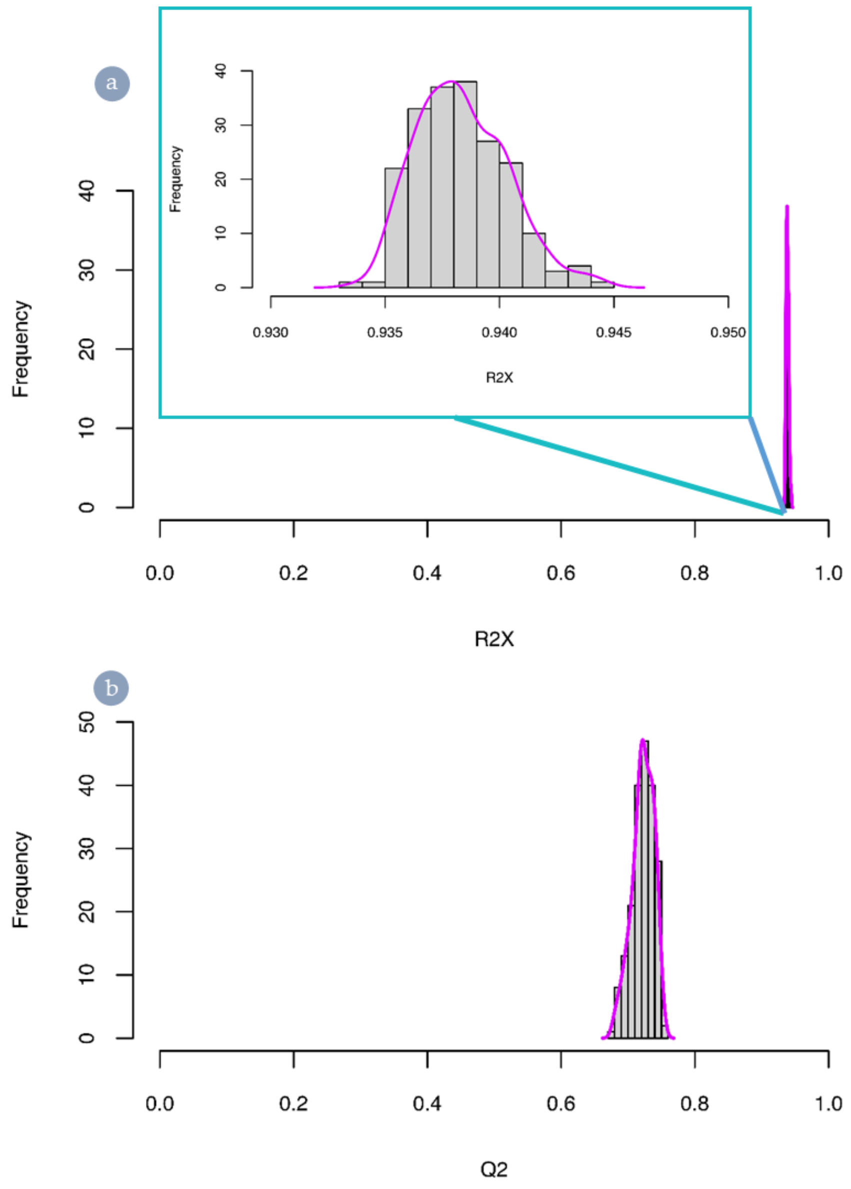

Figure S1: PCA model validation, **(a)** Histogram of R^2 information, showing good fit quality of all permuted models, **(inset)** zoomed-in histogram for clarity, **(b)** histogram depicting Q^2 information.

Table S1: Figures of merit for PCA model analysis.

			Models					
**Number**	**Model**	**Type**	**A**	**N**	**R^2X**	**R^2Y(cum)**	**Q^2**	**Model Content**
1	M1	PCA-X	3	360	0.974	N/A	0.906	All Data
2	M2	PCA-X	3	150	0.985	N/A	0.939	Internals Only (U251 Removed)
3	M3	PCA-X	3	150	0.978	N/A	0.935	Externals Only (U251 Removed)

A = number of principal components; **N** = number of samples in the model; **R^2X** = fraction of the variance of the *X* variables explained by the model; **Q^2** = predictive ability parameter of the model.

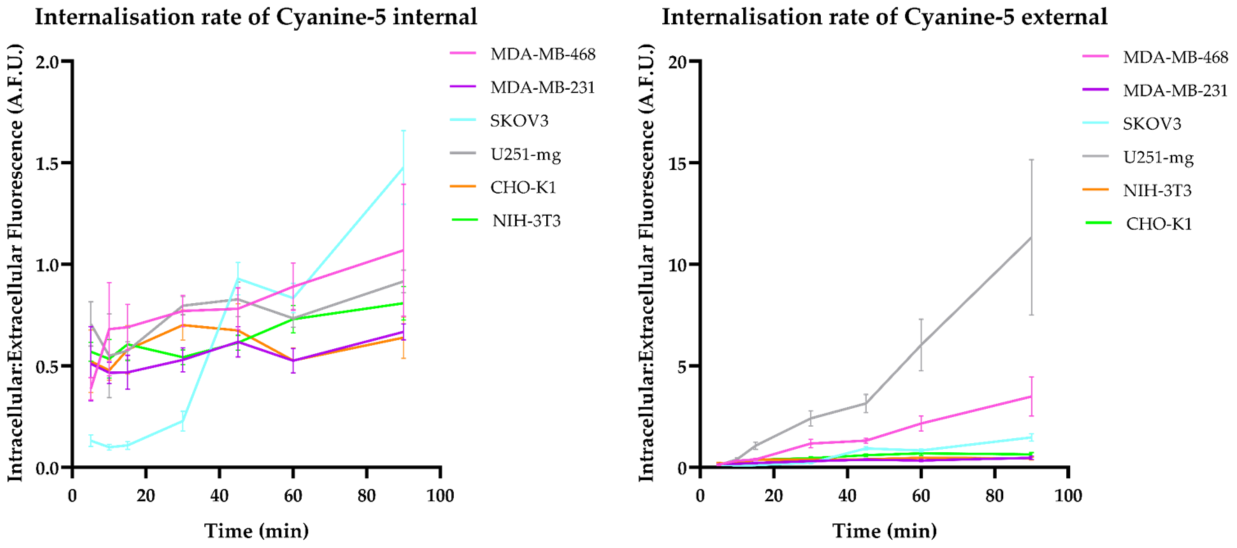

Figure S2: ROI kinetic rate data plotted by HBP analogue, **(a)** Cy5 internal, and **(b)** Cy5 external.

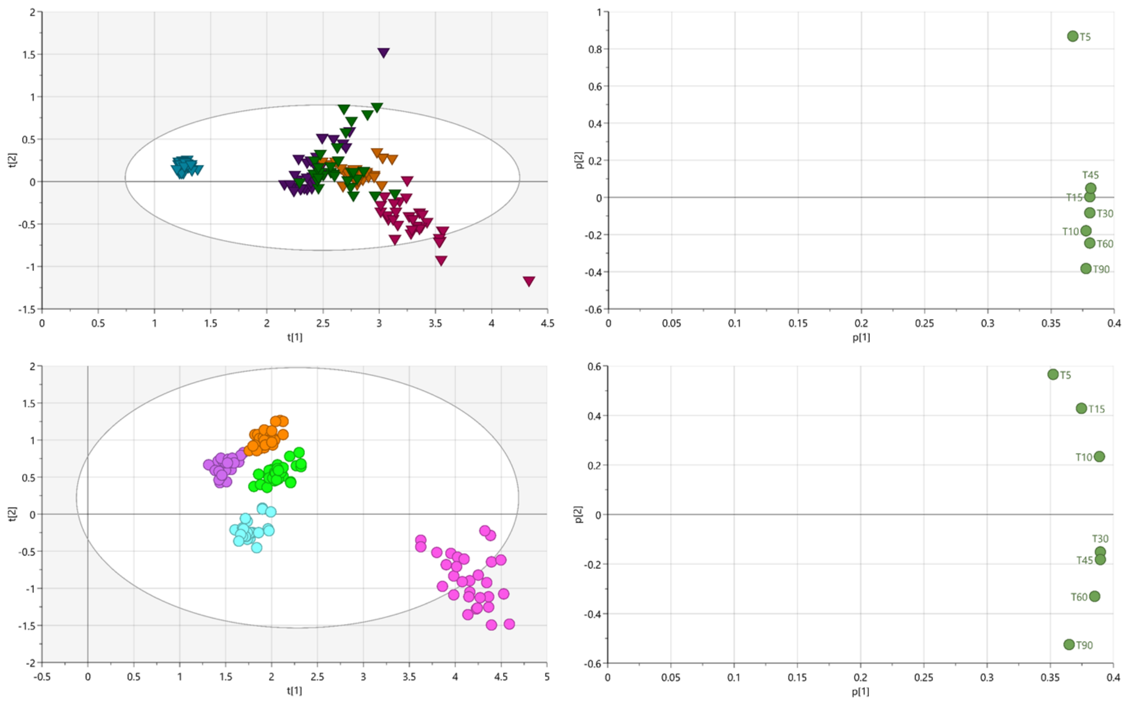

Figure S3: PCA results of U251-mg excluded data, showing separation along t[1] and t[2].

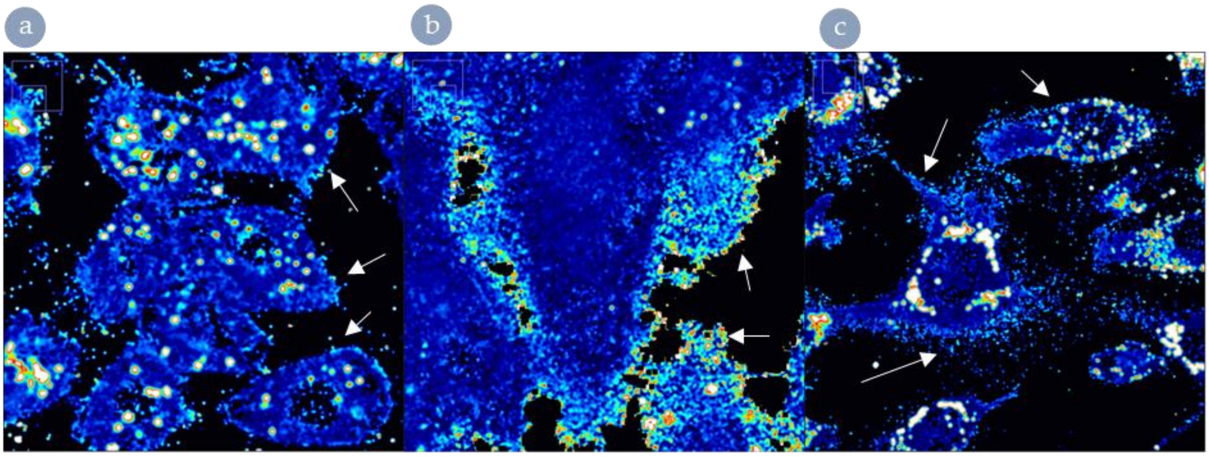

Figure S4: Polar lipid-rich membrane microdomains identified through ratiometric images, **(a)** zoomed-in image of an MDA-MB-468 cell membrane **(b)** zoomed-in image of a U251-mg cell membrane, **(c)** zoomed-in image of an SK-OV-3 cell membrane.
